# Lecanemab and Donanemab as Therapies for Alzheimer's Disease: An Illustrated Perspective on the Data

**DOI:** 10.1523/ENEURO.0319-23.2024

**Published:** 2024-06-28

**Authors:** Alberto J. Espay, Kasper P. Kepp, Karl Herrup

**Affiliations:** ^1^Department of Neurology, James J. and Joan A. Gardner Family Center for Parkinson’s Disease and Movement Disorders, University of Cincinnati, Cincinnati, Ohio 45219; ^2^Section of Biophysical and Biomedicinal Chemistry, Department of Chemistry, Technical University of Denmark, Kongens Lyngby 2800, Denmark; ^3^Department of Neurobiology, University of Pittsburgh, Pittsburgh, Pennsylvania 15261

**Keywords:** aducanumab, Alzheimer's disease, antiamyloid monoclonal antibodies, beta-amyloid, donanemab, lecanemab

## Significance Statement

Treatment of Alzheimer's disease by targeting the antiamyloid beta (Aβ) peptide with immunotherapy has led to Food and Drug Administration approval of several new Aβ monoclonal antibodies. These approvals have come with restrictions, but the uptake of these new therapies in the clinic is expected to increase rapidly, at least in the USA. Hailed as a “breakthrough” by some, there has been stiff countercommentary questioning both safety and efficacy. The authors of this piece have been among those most concerned about the wisdom of releasing these drugs for clinical use. We note that the debate has been thus far largely confined to the clinical literature. With this Social Issues commentary, the authors hope to bring the basic science research community into the discussion.

In 2021, despite investing tens of billions of research dollars, the field of Alzheimer's disease (AD) research was struggling. Since the approval of memantine in 2003 and the extension of donepezil for the treatment of severe AD in 2006 (Feldman et al., 2001), no new treatment for AD had made it to the regulatory approval for nearly 20 years. Worse, efforts to develop treatments based on removing amyloid from the brain had repeatedly failed in late-stage clinical trials. Then, over the succeeding 2 years, three Phase 3 clinical trials of anti-amyloid beta (Aβ) monoclonal antibodies (mAb) were completed, and their results were trumpeted as breakthroughs. First was the Biogen drug, aducanumab (marketed as Aduhelm), followed in quick succession by the Biogen/Eisai drug lecanemab (Leqembi) and the Lilly drug, donanemab. The first two were fast-tracked by the United States Food and Drug Administration (FDA), and lecanemab later received full approval for the treatment of AD. This was the first FDA-approved AD drug since the acetylcholine esterase inhibitor donepezil.

**Figure 1.**
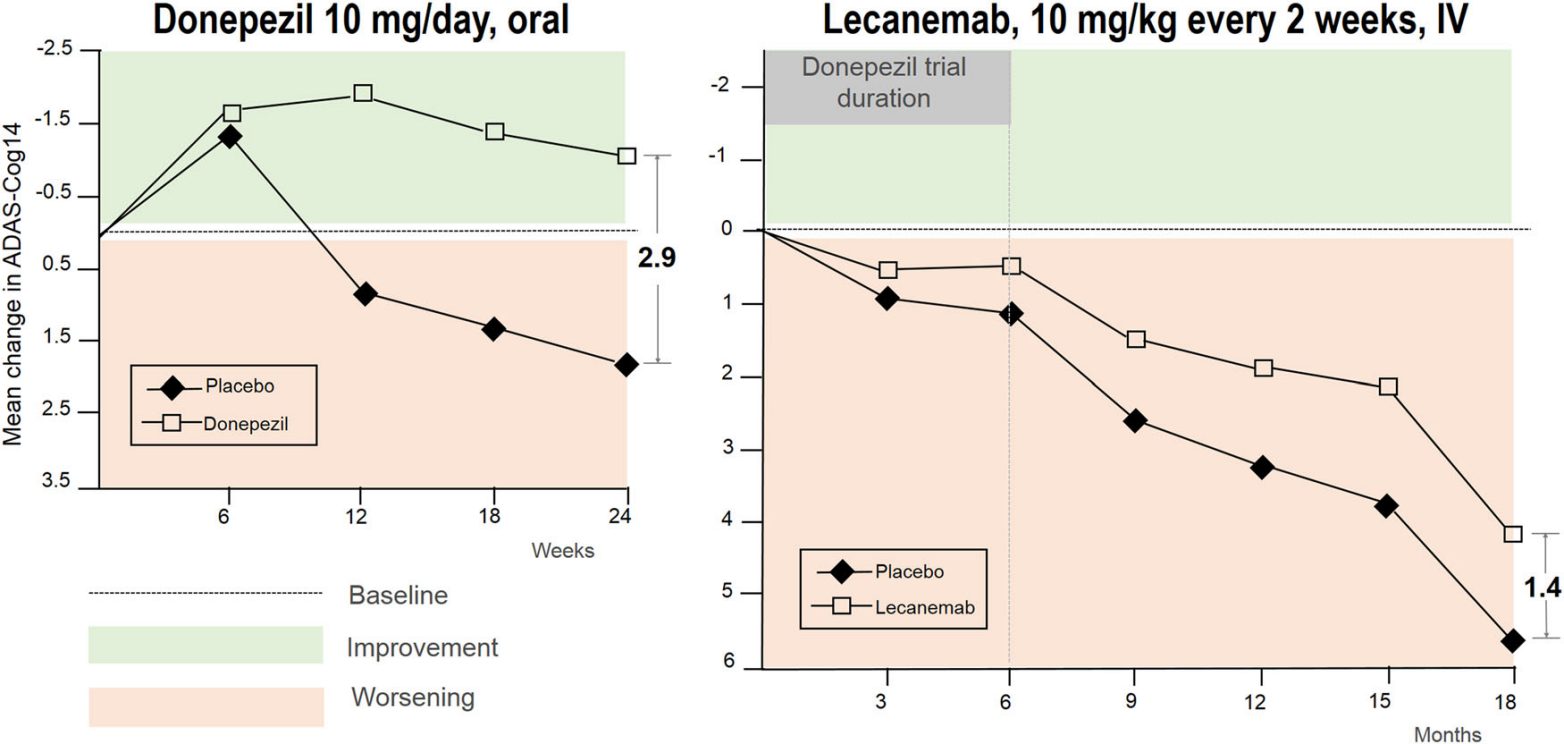
Comparison of the efficacy slopes and absolute changes for donepezil and lecanemab versus placebo. Improvement from the baseline was only observed for donepezil. In both cases, visual divergence in the curves suggested the possibility of greater benefits beyond the short window of the trials. Note the scale of *Y* axis is equal in both studies, facilitating comparison. IV, intravenous. ADAS-Cog, cognitive subscale of the Alzheimer's Disease Assessment Scale; range 0–90, higher means worse. The MCID for this scale is 3 points. Adapted from data in Rogers (1998) and van Dyck et al. (2023).

As exciting as these developments appear, a comparison with the successful donepezil trial completed decades ago provides some context. The slopes between the curves of donepezil and placebo were described as diverging (Fig. 1; Rogers, 1998). This is important because it is often argued that the magnitude of the difference at the end of a clinical trial is less relevant than the situation years later. The hope is that the separation between placebo and drug will continue to grow (Doody et al., 2001). Unfortunately, long-term divergence between treatment and control never materialized for donepezil.

In the recent clinical trials of the mAb, lecanemab, and donanemab, after 18 months of biweekly infusions, the difference between the curves of people on antibody and those on placebo differed by only half the difference found in the donepezil trial after 6 months. For all participants, in both treatment and placebo arms, cognition declined or worsened over the 72 week trial period. While one may hypothesize that continued divergence will continue between the treatment and the placebo arms, in the absence of follow-up studies, evidence for this hope is lacking.

The reports of the clinical trials of lecanemab and donanemab focused on the relative changes between the active and placebo arms. Figures on treatment-induced cognitive changes, expressed as a ratio of the score for the placebo arm (see below), range between 20 and 40%. These seemingly impressive numbers are frequently mentioned, including by the National Institute on Aging (Hodes, 2023). These ratios are a reasonable way to think about the data, but they miss the essential context of the full range of the cognitive scales used. Ratios of effects can be large even with minuscule absolute clinical differences, thus misleading patients and doctors about what the mAb can really do. To provide more complete context, we created visual summaries of the data from the Phase 3 clinical trials of lecanemab (van Dyck et al., 2023) and donanemab (Sims et al., 2023).

## On Efficacy

**Figure 2.**
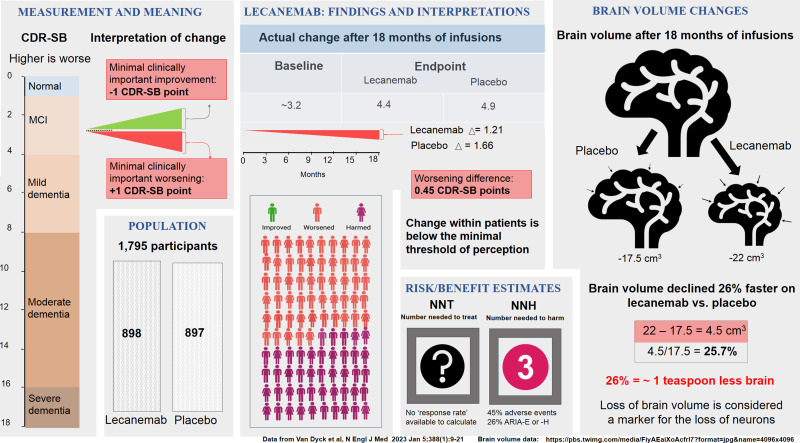
Infographic of the lecanemab pivotal trial. The data are shown to facilitate a comprehensive interpretation of the clinical trial data in the authors’ perspective.

Lecanemab (Fig. 2) is reported to have slowed decline by 27%. This figure was calculated using the Clinical Dementia Rating Scale Sum of Boxes (CDR-SB), an 18-point scale that measures cognitive function (higher is worse). The average participant began with a score of 3.2, the equivalent of mild cognitive impairment. After 18 months, the scores increased (worsened) by 1.66 points to 4.86 in the placebo arm and by 1.21 to 4.41 in the lecanemab arm, i.e., 0.45 points less decline with lecanemab. For a start, it should be noted that 0.45 is arguably less than half the change patients will typically be able to perceive (Lansdall et al., 2023). Also, the Phase 2 trial of lecanemab reported a similar change in decline, but this difference was not statistically significant due in part to a smaller sample size (854 participants; Swanson et al., 2021). Furthermore, in both Phase 2 and 3 trials, the change was only around half the effect reported for donepezil (Birks and Harvey, 2018), which is considered a modest symptomatic treatment.

The formula used by the lecanemab authors was based on the following terms:

$$X=[\mathrm{Le}c_{0}-\mathrm{Le}c_{\Omega }]\;(\mathrm{the}\;\mathrm{absolute}\;\mathrm{value}\;\mathrm{of}\;\mathrm{the}\;\mathrm{lecanemab}\;\mathrm{score}\;\mathrm{change}=1.21),$$

$$Y=[\mathrm{Plc}b_{0}-\mathrm{Plc}b_{\Omega }]\;(\mathrm{the}\;\mathrm{absolute}\;\mathrm{value}\;\mathrm{of}\;\mathrm{the}\;\mathrm{placebo}\;\mathrm{score}\;\mathrm{change}\;=1.66).$$

The percent “improvement” was then calculated as [*X* − *Y*] / *Y*. This is how the 27% effect figure is obtained. This is a perfectly legitimate relative rate calculation and a valuable (but not sufficient) way to think about the data. The relative rate metric informs about the superiority of treatment versus placebo but does not convey how the change relates to a participant's full cognitive capacity. Very small absolute changes can result in very large relative changes, which could be misleading. The preference of relative over absolute changes has led healthcare providers and the public to overestimate concerns about high cholesterol and artificially inflate the magnitude of the benefits of cholesterol-lowering therapy (Diamond and Leaverton, 2023). Relative and absolute metrics are both needed as they supplement each other.

Expressing the data in this way also tacitly assumes that the treatment and placebo curves will continue to separate: for this metric to remain constant, the curves must separate linearly. However, if over time the curves become parallel, as might easily be the case for many patients, then this metric will decline because it divides a constant difference by the larger and larger decline in the placebo group. Thus, values reported early in the treatment will mislead the reader when applied to longer time scales. As an example, after 36 months (double the length of the lecanemab trial), [*X* − *Y*] would need to increase to 0.9 to maintain a 27% “effect.” We propose that this is improbable given the shapes of the published curves.

The Van Dyck method ([*X* − *Y*] / *Y*) thus ignores the important issue of where the participants were relative to the full cognitive scale. One way to approach this is to divide [*X* − *Y*] by the value of the full scale [18 for the CDR-SB; 144 for Integrated Alzheimer's Disease Rating Scale (iADRS)]. This translates to a treatment effect of 2.5% (0.45 / 18) for lecanemab and 2.3% (3.4 / 144) for donanemab. Viewing the data in this way has the advantage of a standardized way of describing the effect size, making it more meaningful to compare different cohorts measured with different scales, yet in some ways it also presents a distorted view. The probability of different scores along the scales is not equal. For example, virtually no subject will score zero on the iADRS or 18 on the CDR-SB. Because of this, the estimate of the treatment effect will be artificially deflated. Thus, these two metrics are incomplete and convey different information and, some would say, different narratives on the efficacy of the drug.

To describe the change due to treatment, while taking into account where the patients were in the progression of their disease, we suggest a third approach: divide the difference between the final scores, [Lec_Ω_ − Plcb_Ω_], by the difference between the initial score of the participants (Lec_0_ or Plcb_0_) and the top score on the scale (144 for iADRS; 0 for CDR-SB). This method has the advantage of accounting for any reversed polarity of cognitive scales (better scores could be higher or lower) and will normalize the scores at any position on the scale. For the lecanemab example, this variation suggests that the treatment led to a 15.6% change over placebo ([4.9 − 4.4] / [3.2 − 0]). All three measures have strengths and weaknesses, and thus discussing them in combination seems warranted in order to better inform treatment decisions.

**Figure 3.**
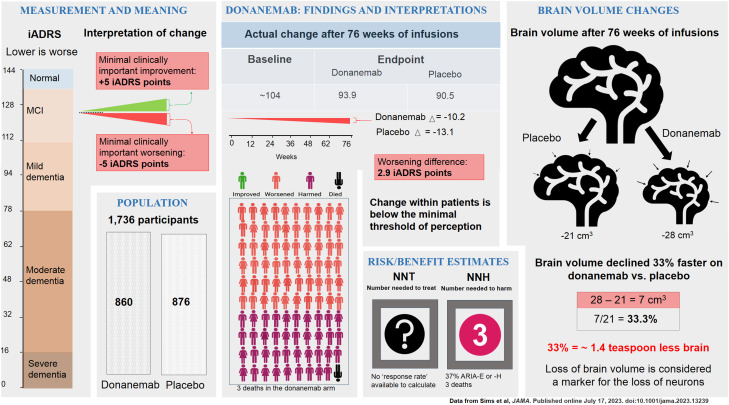
Infographic of the donanemab pivotal trial. The data are shown to facilitate a comprehensive interpretation of the clinical trial data in the authors’ perspective.

If we use the calculations of Sims et al., donanemab (Fig. 3) slowed decline by 33% in the “ideal” subgroup of “low/medium tau” participants. The trial used as outcome measure the iADRS, a 144-point composite scale measuring cognition plus activities of daily living items (lower is worse). The average participant began with a score of 104 (mild dementia). After 76 weeks, the scores decreased by −13.1 points to 90.5 in the placebo arm and by 10.2 points to 93.9 in the donanemab arm—3.4 points lesser decline. The 33% figure comes from dividing [*X* − *Y*] by *Y* (3.4 / 10.2). Here again, other ways of viewing the data provide much needed context. If we use the full scale as the denominator instead of *Y*, the effect is 2.4% (3.4 / 144). If we use the third method, donanemab's effect would be 8.5% ([93.9 − 90.5] / [104 − 144]) in absolute terms, on the relevant part of the scale. These numbers in context are useful for a full appreciation of the data.

All ways of describing the lecanemab and donanemab data are legitimate but incomplete. For the patient, the salient question is: Are these effects meaningful? Considerable work has gone into defining what is known as the “minimal clinically important difference” (MCID), the smallest change a patient can perceive (Cook, 2008). While the MCID was not designed to provide a metric to evaluate efficacy of group-level differences in clinical trials, we lack individual-level data to determine what the changes might mean for any one person (Lansdall et al., 2023). This caveat notwithstanding, for iADRS, this number is 5 for patients with mild cognitive impairment and 9 for patients with mild dementia (Wessels et al., 2022); for CDR-SB, the number is 1.0 for mild cognitive impairment and 1.6 for mild dementia. Average changes of ∼3 on the iADRS and ∼0.5 on the CDR-SB represent differences imperceptible to patients, their families, and their physicians, no matter what percentage we use to describe the difference.

## On Safety

With a disease as devastating as AD, even a small effect size should be considered by clinicians if treatments were safe. However, these mAb are not safe. In both trials, adverse events afflicted sizable numbers of participants. With lecanemab, 45% of the participants had treatment-related adverse events, with nearly one in four patients developing brain swelling and/or bleeding, which proved to be severe in some persons. Severe bleeding occurred to a greater extent compared with placebo (five vs one in the lecanemab trial; seven vs two in the donanemab trial), including three fatal cases. With donanemab, 89% of patients had treatment-related adverse events and more than one in three patients developed brain swelling and/or bleeding. Brain swelling is a major contributor to the acceleration of brain atrophy, a feature of most mAb (Alves et al., 2023).

In evaluating a therapy, two useful numbers are “the number needed to harm” (NNH) and “the number needed to treat” (NNT). The NNH is the number of patients that need to receive the treatment for one to experience an adverse outcome (Andrade, 2015). When accounting for all toxic effects, that number is ∼3 for both lecanemab and donanemab. The NNT is the number of patients that need to receive the treatment for one to experience a beneficial response. As discussed above, no individual patient-level data was reported during these trials, making this number impossible to accurately calculate. We note as well that due to the assumptions that must be made (discussed above), it is even hard to estimate NNT from the average data. Thus, we have marked the NNT with a “?” sign in Figures 2 and 3. We caution that as these treatments enter the real world of clinical practice, people may be exposed to substantial risks without the benefit of the rigorous screening and careful monitoring that was part of the Phase 3 trials, potentially magnifying the harm.

In summary, the new mAb do not improve cognition but rather reduce the decline by an arguably smaller magnitude than donepezil and below the individual threshold of perception for most patients, carrying a worrisome safety profile. The advantage of these drugs seems to be related more to the theoretical possibility that they are disease modifying (in keeping with the prevailing framework of amyloid toxicity), rather than in their actual risk/cost–benefit trade-off. Before these expensive drugs are administered broadly, both the safety profile of subgroups and the efficacy relative to the benchmark drug donepezil should ideally be tested. If this does not happen, we risk adopting a potentially less effective drug with significant potential harm over the benchmark drug on the market, donepezil. Excess deaths occur with mAb infusions along with frequent brain swelling and bleeding. This concern is all the more acute given the acceleration of brain volume loss they induce (Alves et al., 2023). We leave a detailed discussion of the costs of these therapies for another day. We would note, however, that a substantial impact on our healthcare networks would be unavoidable if even only 10% of at-risk patients were to be subjected to these infusions.

The small and uncertain benefits, the worrisome and poorly understood risks, and the very high costs of treatment suggest that these drugs are promoted largely out of theoretical rather than practical benefits. For patients and society—both of whom bear the costs of this treatment—caveat emptor.

## Synthesis

Reviewing Editor: Christie Fowler, University of California Irvine Decisions are customarily a result of the Reviewing Editor and the peer reviewers coming together and discussing their recommendations until a consensus is reached. When revisions are invited, a fact-based synthesis statement explaining their decision and outlining what is needed to prepare a revision will be listed below. The following reviewer(s) agreed to reveal their identity: Joshua Grill, Michelle Jin.

In reviewing the revised version of the manuscript, significant concerns remain. Both the infographic and third statistic are noted as flawed. Additional points that need to be addressed to be considered for publication include:

Reviewer 1:

There remain some careless errors and flawed arguments in the manuscript. Given that a main point of the article seems to be to suggest that the authors of the primary papers describing trials of lecanemab and donanemab were biased in their approach to presenting data, the authors could do a better job to use careful wording and scientific approaches to support their claims.

- The last approval in AD was not donepezil, it was memantine.

- The divergence of slopes is most often offered as supportive of disease-modifying treatment effects and indeed it is suggested that this difference would grow over time for a truly disease modifying therapy. However, few (albeit some) argue that donepezil is a disease-modifying therapy and it is clear that the biological effects of the monoclonal antibodies are highly distinct from the mechanisms and effects of the cholinesterase inhibitors. Furthermore, the "hoped for divergence" is a divergence from a placebo group and therefore requires longer trials. So it is unclear what the basis is for the statement "the hoped for divergence never materialized."

- The authors should take care to use less subjective language, such as "it is often argued that the magnitude of the difference at the end of a clinical 18 trial is less relevant than the situation years later"

- The figure should really use similar x and y axes if the goal is a fair comparison.

- The statement on page 3 "there is no definitive evidence for this, and the case of donepezil suggests that this is not generally the case" is not adequately evidence based. Do the authors wish to generalize one drug with mechanism to all other drugs of other mechanisms?

- The author's comments on % difference from placebo are well-argued. However, they need to offer further justification some of the other assessments, such as calling people "worsened" in the infographics. Patients with neurodegenerative disease worsen over time, so to assign this worsening to the treatment (when the goal is slowing worsening) is unfair. Similarly, it is curious that the infographic suggests they can assess "improvement." This would seem to require patient-level data to quantify the proportion whose scores at end of study were improved relative to baseline (recent presentations at CTAD suggests that some patients may have actually fallen into this category). Did they have such data?

- The "on efficacy" sections seem fine. The authors propose alternative arithmetic approaches that are their opinions but seem justified.

- On safety, they fail to consider the difference from placebo when citing overall AE rates. Older patients with neurodegenerative disease will experience AEs, particularly for long trials with many visits (when investigators inquire about AEs). So to focus on one group is shortsighted and misleading. The relationship between brain swelling and volume loss is unproven. The authors are referred to this thoughtful commentary: https://pubmed.ncbi.nlm.nih.gov/36973045/

- "As no patients improved during these trials, this number cannot be calculated." Do the authors have patient-level data?

Reviewer 2:

In this resubmission, the authors present a graphical perspective on the outcomes of the phase III RCT for lecanemab and donanemab, two disease modifying monoclonal antibodies developed for treating mild cognitive impairment (MCI) and Alzheimer's patients with mild dementia. Strengths of the article include an additional qualitative comparison with previous donepezil trials which provide perspective, and a more thorough exploration of three different metrics for interpreting the effect sizes of these treatments on cognition. For the third metric, the authors present a sound justification to scale treatment effect against the cognitive baseline of the initial group. This presents a novel way to view the effects and perhaps an easier verbal framework for patients to understand effects- i.e., "This drug results in 14% less worsening of cognition relative to your current deficits." However, there remains a major error with the calculations that still does not account for reverse polarity of the iADRS scale, resulting in a flawed interpretation of the third metric they present. These issues should be corrected before further consideration. However, the perspective the authors present remains timely and addresses a larger question of how treatment effects of these monoclonal antibodies should be framed in context of actual clinical improvement. For this reason, the perspective the authors present would be valuable to the readers of eNeuro.

Major:

1) The third metrics is susceptible to the same arithmetic distortion as presented in the original submission if the cognitive scale is descending, as in the iADRS. To illustrate how, let's take two scenarios and contrast what happens if we use the CDS-SB scale vs the iADRS scale:

In the first scenario, the baseline cognitive status is good (a high value relative to the iADRS scale, a small value relative to the CDS-SB scale) and the treatment effect remains fixed (represented as E). In the second the baseline cognitive status is poor (a low value relative to the iADRS scale, a high value relative to the CDS-SB) and the treatment effect remains fixed.

CDS-SB:

Scenario 1: E/(low CDS-SB score) = higher effect proportion

Scenario 2: E/(high CDS-SB score) = lower effect proportion

This make sense as treatment effect as proportion of baseline cognitive deficit should be lower if cognition was initially poor.

For iADRS:

Scenario 1: E/ (high iADRS score) = lower effect proportion

Scenario 2: E/ (low iADRS score) = higher effect proportion

You can see here, it does not make sense that if baseline cognitive status was poor that the effect size should be higher than if the baseline cognitive status was high. Let's say the 3.1% effect size for metric 3 quoted by the authors represents scenario 1 (3.4/105 baseline). If instead the baseline started extremely poor at 10, then 3.4/10=0.34=34% slowing of decline. This does not make sense. I suggest the following metric would better accomplish what the authors are trying to convey:

Abs(Antibody arm final score - Placebo arm final score) / Abs(Group baseline score - perfect score)

This would account for any reversed polarity of cognitive scales, and should normalize the scores, no matter how wide the range of the scale. This makes this metric more useful for comparing outcomes for donanemab and lecanemab directly. As a gut check, using this metric for the donanemab scale yields 8.5% cognitive slowing: abs(93.9-90.5)/ abs(104-144) = 0.085 = 8.5%. Given the similarity between the two trials, this higher effect size makes more sense and is more similar to the 14% cognitive slowing effect of lecanemab reported by the authors.

2) In Figure 3 the Donanemab and Placebo arm final cognitive scores are still switched. See under "Change between groups".

Minor:

3) Lines 54-55: The presentation of the equations is slightly confusing, as I initially read the em dash as a subtraction sign. I would suggest the following:

X = [Lec0 - LecΩ] (the absolute value of the lecanemab score change) = 1.21

4) Lines 88-90 states "This provides information on the absolute change in the context of the actual scale, and thus the disease stage." This statement is confusing to me, as the change in cognitive scores against the actual scale is better represented with metric two, where the difference in cognitive scores is divided by the full range of the cognitive scales. Metric three instead seems to provides information on the relative cognitive change in the context of the initial disease state. I would rephrase this.

5) For Figures 2 & 3, it is confusing to report the absolute final score of the treatment and placebo arms as "Change between groups." It may be helpful to rephase as "Final score."
